# *catena*-Poly[[tetra­kis­(hexa­methyl­phospho­ramide-κ*O*)bis­(nitrato-κ^2^ *O*,*O*′)lutetium(III)] [silver(I)-di-μ-sulfido-tungstate(VI)-di-μ-sulfido]]

**DOI:** 10.1107/S1600536812020983

**Published:** 2012-05-16

**Authors:** Jinfang Zhang

**Affiliations:** a Molecular Materials Research Center, Scientific Research Academy, School of Chemistry and Chemical Engineering, Jiangsu University, Zhenjiang 212013, People’s Republic of China

## Abstract

In the title compound, {[Lu(NO_3_)_2_(C_6_H_18_N_3_OP)_4_][AgWS_4_]}_*n*_, hexa­methyl­phospho­ramide (hmp), tetra­thio­tungstate, silver iodide and lutetium nitrate were self-assembled, forming an anionic {[AgWS_4_]^−^}_*n*_ chain extending parallel to [001]. The Lu^III^ atom in the cation is coordinated by eight O atoms from two nitrate groups and four hmp ligands in a distorted square-anti­prismatic geometry. Together with the two nitrate groups, the cation is monovalent, which leads to the anionic chain having a [WS_4_Ag] repeat unit. The polymeric anionic chain has a distorted linear configuration with W—Ag—W and Ag—W—Ag angles of 161.66 (2) and 153.503 (12)°, respectively. The title complex is isotypic with the Y, Yb, Eu, Nd, La, Dy and Sm analogues.

## Related literature

For one-dimensional Mo(W)/S/Ag anionic polymers, see: Niu *et al.* (2004 ▶); Zhang, Meng *et al.* (2010 ▶). For the structures of isotypic Y, Yb, Eu, Nd, La, Dy and Sm complexes, see: Zhang, Cao *et al.* (2007 ▶); Zhang (2010 ▶, 2011*a* ▶,*b* ▶); Cao *et al.* (2007 ▶); Zhang, Qian *et al.* (2007 ▶); Tang, Zhang & Zhang (2008 ▶); Tang, Zhang, Zhang & Lu (2008 ▶).
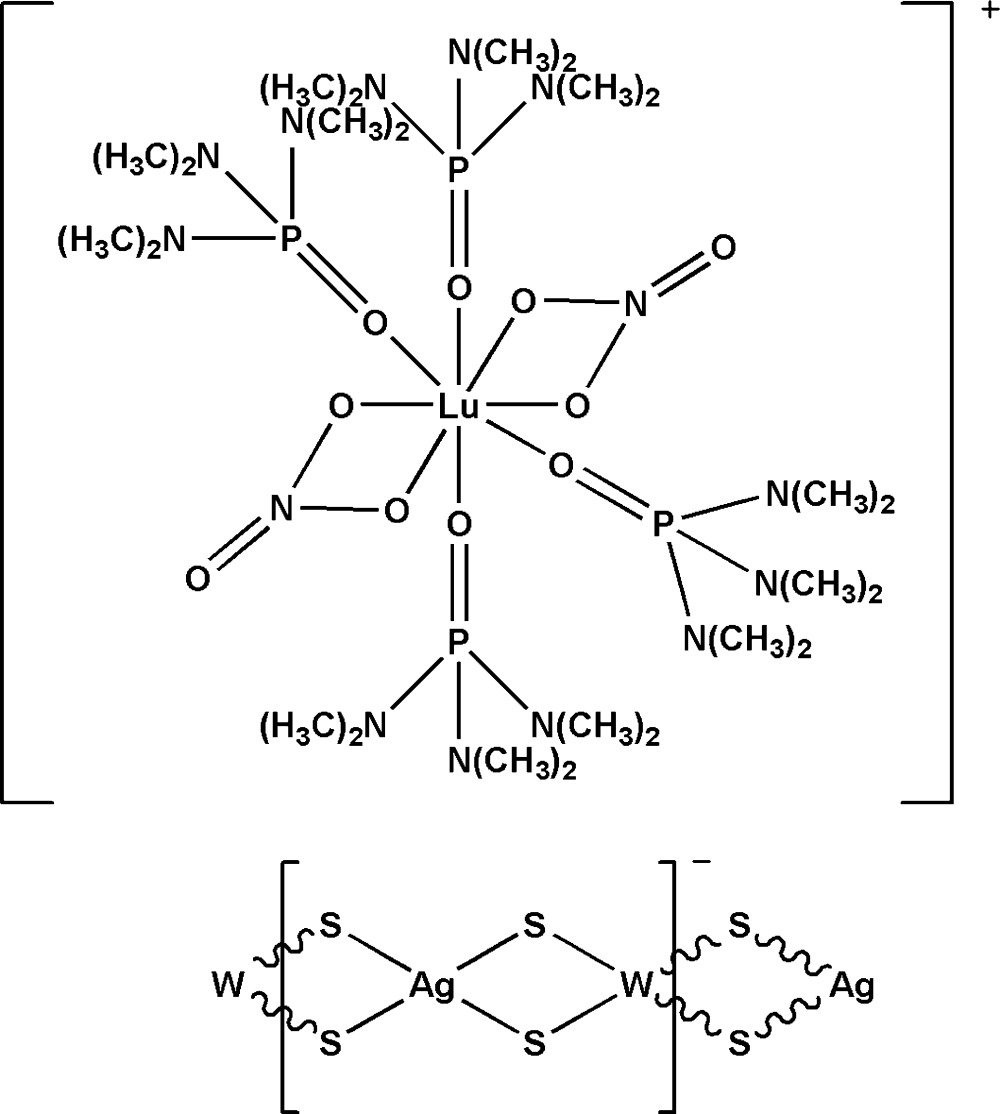


## Experimental

### 

#### Crystal data

[Lu(NO_3_)_2_(C_6_H_18_N_3_OP)_4_][AgWS_4_]
*M* *_r_* = 1435.80
Monoclinic, 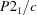

*a* = 15.714 (3) Å
*b* = 29.505 (6) Å
*c* = 11.378 (2) Å
β = 90.54 (3)°
*V* = 5275.1 (17) Å^3^
*Z* = 4
Mo *K*α radiation
μ = 4.74 mm^−1^
*T* = 293 K
0.21 × 0.15 × 0.12 mm

#### Data collection

Rigaku Saturn724+ diffractometer
Absorption correction: multi-scan (*CrystalClear*; Rigaku, 2008 ▶) *T*_min_ = 0.758, *T*_max_ = 1.000
26979 measured reflections
9613 independent reflections
8961 reflections with *I* > 2σ(*I*)
*R*_int_ = 0.038

#### Refinement

*R*[*F* ^2^ > 2σ(*F* ^2^)] = 0.043
*wR*(*F* ^2^) = 0.089
*S* = 1.09
9613 reflections
532 parameters
H-atom parameters constrained
Δρ_max_ = 1.35 e Å^−3^
Δρ_min_ = −1.46 e Å^−3^

### 

Data collection: *CrystalClear* (Rigaku, 2008 ▶); cell refinement: *CrystalClear*; data reduction: *CrystalClear*; program(s) used to solve structure: *SHELXTL* (Sheldrick, 2008 ▶); program(s) used to refine structure: *SHELXTL*; molecular graphics: *SHELXTL*; software used to prepare material for publication: *SHELXTL*.

## Supplementary Material

Crystal structure: contains datablock(s) I, global. DOI: 10.1107/S1600536812020983/rz2754sup1.cif

Structure factors: contains datablock(s) I. DOI: 10.1107/S1600536812020983/rz2754Isup2.hkl

Additional supplementary materials:  crystallographic information; 3D view; checkCIF report

## Figures and Tables

**Fig. 1.**
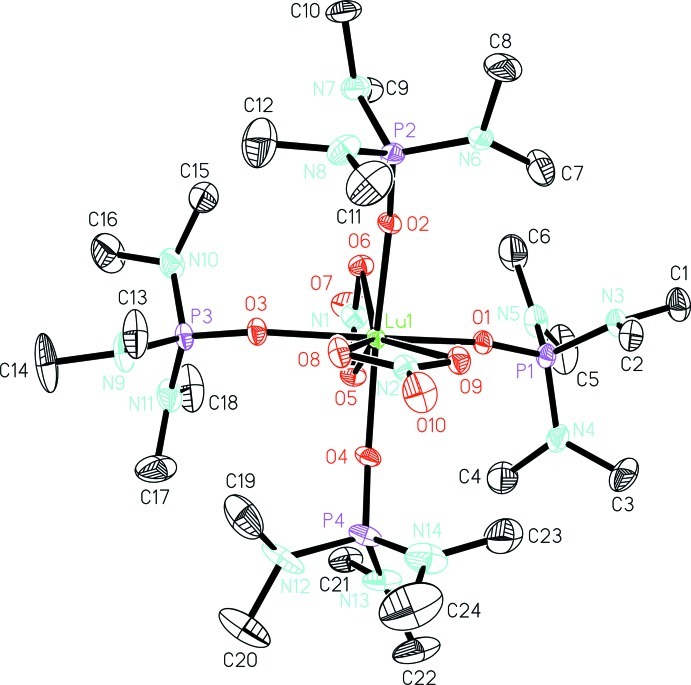
The molecular structure of the cation in the title compound, with 30% probability displacement ellipsoids. All H atoms have been omitted.

**Fig. 2.**
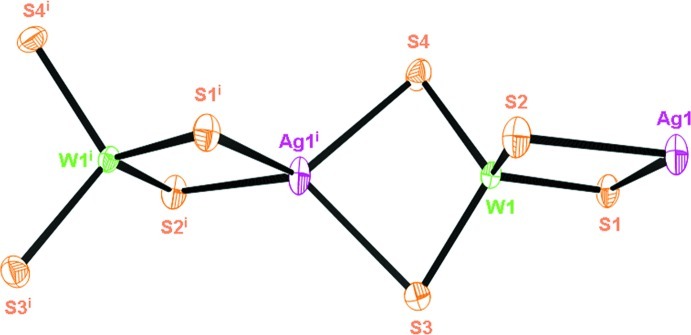
The molecular structure of a portion of the anionic chain in the title compound, with 30% probability displacement ellipsoids, Symmetry code: (i) x, -y + 1/2, z - 1/2.

## supplementary crystallographic information

### Comment

One-dimensional Mo(W)/S/Ag anionic polymers have attracted much attention for their intriguing structures (Niu *et al.*, 2004 and Zhang, Meng *et al.* 2010). Different solvent-coordinated rare-earth cations proved effective to obtain various configurations of anionic chains (Niu *et al.*, 2004). The title compound {[Lu(hmp)_4_(NO_3_)_2_][WS_4_Ag]}_n_ (hmp = hexamethylphosphoramide) with a wave-like anionic chain was prepared by following such route using Lu(III)-hmp complex as counterion.

The title complex is isostructural with Y (Zhang, Cao *et al.*, 2007 and Zhang *et al.*, 2011*a*), Yb (Cao *et al.*, 2007), Eu (Zhang, Qian *et al.*, 2007), Nd (Tang, Zhang & Zhang, 2008), La (Tang, Zhang, Zhang & Lu, 2008), Dy(Zhang *et al.*, 2010) and Sm (Zhang *et al.*, 2011*b*) isomorphs. Lu^3+^ in the cation is coordinated by eight O atoms from two nitrate and four hmp ligands. In possession of two nitrate ligands, the cation in the title compound is univalent (Fig. 1), which leads to an anionic chain with a univalent repeat unit. As illustrated in Fig. 2, the anionic chain in the title compound has a distorted linear configuration with W—Ag—W and Ag—W—Ag angles of 161.66 (2) and 153.503 (12)° respectively.

### Experimental

1 mmol AgI was added to a solution of [NH_4_]_2_WS_4_ (1 mmol in 10 ml hmp) with thorough stirring for 30 minutes. The solution underwent additional stirring for two minute after 0.5 mmol Lu(NO_3_)_3_.6H_2_O was added. After filtration the orange filtrate was carefully laid on the surface with 15 ml *i*-PrOH. Orange block crystals were obtained after about one week.

### Refinement

H atoms were positioned geometrically and refined with a riding model, with *U*_iso_(H) = 1.5*U*_eq_/C) and C—H = 0.96 Å.

### Crystal data

**Table:**

[Lu(NO_3_)_2_(C_6_H_18_N_3_OP)_4_][AgWS_4_]	*F*(000) = 2840
*M**_r_* = 1435.80	*D*_x_ = 1.808 Mg m^−^^3^
Monoclinic, *P*2_1_/*c*	Mo *K*α radiation, λ = 0.71073 Å
Hall symbol: -P 2ybc	Cell parameters from 14630 reflections
*a* = 15.714 (3) Å	θ = 3.0–29.1°
*b* = 29.505 (6) Å	µ = 4.74 mm^−^^1^
*c* = 11.378 (2) Å	*T* = 293 K
β = 90.54 (3)°	Block, orange
*V* = 5275.1 (17) Å^3^	0.21 × 0.15 × 0.12 mm
*Z* = 4	

### Data collection

**Table:**

Rigaku Saturn724+ diffractometer	9613 independent reflections
Radiation source: fine-focus sealed tube	8961 reflections with *I* > 2σ(*I*)
Graphite monochromator	*R*_int_ = 0.038
ω scans	θ_max_ = 25.4°, θ_min_ = 3.0°
Absorption correction: multi-scan (*CrystalClear*; Rigaku, 2008)	*h* = −18→18
*T*_min_ = 0.758, *T*_max_ = 1.000	*k* = −28→35
26979 measured reflections	*l* = −11→13

### Refinement

**Table:**

Refinement on *F*^2^	Primary atom site location: structure-invariant direct methods
Least-squares matrix: full	Secondary atom site location: difference Fourier map
*R*[*F*^2^ > 2σ(*F*^2^)] = 0.043	Hydrogen site location: inferred from neighbouring sites
*wR*(*F*^2^) = 0.089	H-atom parameters constrained
*S* = 1.09	*w* = 1/[σ^2^(*F*_o_^2^) + (0.025*P*)^2^ + 23.7331*P*] where *P* = (*F*_o_^2^ + 2*F*_c_^2^)/3
9613 reflections	(Δ/σ)_max_ = 0.001
532 parameters	Δρ_max_ = 1.35 e Å^−^^3^
0 restraints	Δρ_min_ = −1.46 e Å^−^^3^

### Special details

**Table:**

Geometry. All e.s.d.'s (except the e.s.d. in the dihedral angle between two l.s. planes) are estimated using the full covariance matrix. The cell e.s.d.'s are taken into account individually in the estimation of e.s.d.'s in distances, angles and torsion angles; correlations between e.s.d.'s in cell parameters are only used when they are defined by crystal symmetry. An approximate (isotropic) treatment of cell e.s.d.'s is used for estimating e.s.d.'s involving l.s. planes.
Refinement. Refinement of *F*^2^ against ALL reflections. The weighted *R*-factor *wR* and goodness of fit *S* are based on *F*^2^, conventional *R*-factors *R* are based on *F*, with *F* set to zero for negative *F*^2^. The threshold expression of *F*^2^ > σ(*F*^2^) is used only for calculating *R*-factors(gt) *etc*. and is not relevant to the choice of reflections for refinement. *R*-factors based on *F*^2^ are statistically about twice as large as those based on *F*, and *R*- factors based on ALL data will be even larger.

### Fractional atomic coordinates and isotropic or equivalent isotropic displacement parameters (Å^2^)

**Table:**

	*x*	*y*	*z*	*U*_iso_*/*U*_eq_	
Lu1	0.238835 (16)	0.082664 (9)	0.82761 (3)	0.02424 (8)	
P1	0.02402 (11)	0.13199 (6)	0.81901 (17)	0.0313 (4)	
P2	0.20044 (12)	−0.02970 (6)	0.70329 (18)	0.0335 (4)	
P3	0.45726 (11)	0.09618 (6)	0.73055 (18)	0.0328 (4)	
P4	0.29341 (14)	0.14657 (7)	1.09306 (19)	0.0420 (5)	
O1	0.1061 (3)	0.10628 (15)	0.8222 (4)	0.0312 (11)	
O2	0.2079 (3)	0.01909 (14)	0.7318 (4)	0.0313 (11)	
O3	0.3745 (3)	0.08059 (15)	0.7786 (5)	0.0346 (12)	
O4	0.2722 (3)	0.12599 (15)	0.9777 (4)	0.0337 (11)	
O5	0.2536 (3)	0.15769 (15)	0.7411 (5)	0.0356 (12)	
O6	0.2249 (3)	0.10224 (15)	0.6230 (4)	0.0353 (11)	
O7	0.2285 (4)	0.1716 (2)	0.5563 (5)	0.0651 (18)	
O8	0.3020 (3)	0.02779 (15)	0.9615 (5)	0.0366 (12)	
O9	0.1691 (3)	0.04180 (16)	0.9851 (5)	0.0360 (12)	
O10	0.2374 (4)	0.00153 (19)	1.1157 (5)	0.0559 (16)	
N1	0.2363 (4)	0.1452 (2)	0.6380 (6)	0.0401 (15)	
N2	0.2366 (4)	0.02294 (19)	1.0234 (6)	0.0357 (14)	
N3	−0.0539 (3)	0.0967 (2)	0.8464 (6)	0.0362 (14)	
N4	0.0232 (4)	0.1731 (2)	0.9157 (6)	0.0478 (18)	
N5	0.0093 (4)	0.1550 (2)	0.6918 (6)	0.0500 (18)	
N6	0.1064 (4)	−0.0476 (2)	0.7424 (6)	0.0477 (18)	
N7	0.2218 (4)	−0.0359 (2)	0.5618 (6)	0.0407 (15)	
N8	0.2662 (4)	−0.0648 (2)	0.7683 (6)	0.0469 (17)	
N9	0.5329 (4)	0.0698 (2)	0.8016 (6)	0.0492 (18)	
N10	0.4550 (4)	0.0869 (2)	0.5876 (6)	0.0508 (18)	
N11	0.4832 (4)	0.1491 (2)	0.7387 (7)	0.054 (2)	
N12	0.3944 (5)	0.1388 (3)	1.1227 (8)	0.073 (3)	
N13	0.2725 (5)	0.2007 (2)	1.0903 (6)	0.056 (2)	
N14	0.2394 (6)	0.1242 (3)	1.1946 (7)	0.070 (2)	
C1	−0.1417 (5)	0.1059 (3)	0.8091 (8)	0.054 (2)	
H1A	−0.1778	0.0814	0.8332	0.081*	
H1B	−0.1442	0.1088	0.7251	0.081*	
H1C	−0.1608	0.1336	0.8445	0.081*	
C2	−0.0450 (5)	0.0644 (3)	0.9441 (7)	0.045 (2)	
H2A	−0.0953	0.0462	0.9489	0.068*	
H2B	−0.0371	0.0808	1.0163	0.068*	
H2C	0.0034	0.0453	0.9309	0.068*	
C3	−0.0461 (6)	0.1832 (3)	0.9949 (9)	0.063 (3)	
H3A	−0.0314	0.2090	1.0422	0.095*	
H3B	−0.0560	0.1575	1.0449	0.095*	
H3C	−0.0967	0.1896	0.9500	0.095*	
C4	0.0899 (6)	0.2078 (3)	0.9141 (10)	0.073 (3)	
H4A	0.0799	0.2294	0.9754	0.109*	
H4B	0.0891	0.2229	0.8394	0.109*	
H4C	0.1444	0.1937	0.9263	0.109*	
C5	−0.0347 (6)	0.1988 (4)	0.6740 (11)	0.092 (4)	
H5A	−0.0367	0.2058	0.5917	0.137*	
H5B	−0.0043	0.2222	0.7152	0.137*	
H5C	−0.0916	0.1967	0.7037	0.137*	
C6	0.0217 (6)	0.1284 (5)	0.5869 (9)	0.089 (4)	
H6A	0.0103	0.1467	0.5189	0.133*	
H6B	−0.0164	0.1029	0.5872	0.133*	
H6C	0.0794	0.1178	0.5849	0.133*	
C7	0.0349 (5)	−0.0171 (3)	0.7493 (10)	0.063 (3)	
H7A	−0.0147	−0.0336	0.7725	0.095*	
H7B	0.0250	−0.0035	0.6738	0.095*	
H7C	0.0470	0.0062	0.8061	0.095*	
C8	0.0812 (7)	−0.0950 (3)	0.7221 (9)	0.076 (3)	
H8A	0.0241	−0.0995	0.7490	0.114*	
H8B	0.1192	−0.1147	0.7645	0.114*	
H8C	0.0840	−0.1016	0.6396	0.114*	
C9	0.1964 (6)	−0.0015 (3)	0.4784 (8)	0.060 (2)	
H9A	0.2138	−0.0103	0.4011	0.090*	
H9B	0.2228	0.0267	0.4991	0.090*	
H9C	0.1357	0.0019	0.4796	0.090*	
C10	0.2270 (6)	−0.0819 (3)	0.5112 (8)	0.061 (3)	
H10A	0.2395	−0.0798	0.4290	0.091*	
H10B	0.1737	−0.0972	0.5214	0.091*	
H10C	0.2714	−0.0986	0.5505	0.091*	
C11	0.2564 (7)	−0.0811 (3)	0.8850 (10)	0.081 (3)	
H11A	0.3029	−0.1010	0.9044	0.121*	
H11B	0.2037	−0.0974	0.8908	0.121*	
H11C	0.2561	−0.0560	0.9387	0.121*	
C12	0.3551 (6)	−0.0675 (4)	0.7344 (12)	0.093 (4)	
H12A	0.3837	−0.0896	0.7823	0.139*	
H12B	0.3816	−0.0384	0.7454	0.139*	
H12C	0.3586	−0.0761	0.6533	0.139*	
C13	0.5197 (5)	0.0278 (3)	0.8603 (10)	0.071 (3)	
H13A	0.5721	0.0181	0.8964	0.107*	
H13B	0.5010	0.0054	0.8046	0.107*	
H13C	0.4772	0.0316	0.9195	0.107*	
C14	0.6230 (5)	0.0792 (4)	0.7778 (10)	0.092 (4)	
H14A	0.6581	0.0604	0.8274	0.138*	
H14B	0.6349	0.1105	0.7938	0.138*	
H14C	0.6348	0.0727	0.6969	0.138*	
C15	0.4059 (6)	0.0484 (3)	0.5418 (9)	0.060 (2)	
H15A	0.4108	0.0473	0.4578	0.090*	
H15B	0.3472	0.0520	0.5625	0.090*	
H15C	0.4276	0.0208	0.5752	0.090*	
C16	0.5274 (7)	0.0983 (4)	0.5151 (9)	0.092 (4)	
H16A	0.5151	0.0905	0.4347	0.138*	
H16B	0.5765	0.0817	0.5417	0.138*	
H16C	0.5386	0.1302	0.5208	0.138*	
C17	0.5150 (8)	0.1682 (4)	0.8498 (10)	0.099 (5)	
H17A	0.5273	0.1998	0.8393	0.148*	
H17B	0.5660	0.1526	0.8735	0.148*	
H17C	0.4726	0.1647	0.9094	0.148*	
C18	0.4520 (6)	0.1835 (3)	0.6577 (12)	0.084 (4)	
H18A	0.4752	0.2125	0.6795	0.126*	
H18B	0.3910	0.1848	0.6608	0.126*	
H18C	0.4693	0.1761	0.5793	0.126*	
C19	0.4350 (6)	0.0961 (4)	1.1099 (11)	0.086 (4)	
H19A	0.4938	0.0986	1.1328	0.129*	
H19B	0.4312	0.0865	1.0294	0.129*	
H19C	0.4075	0.0742	1.1590	0.129*	
C20	0.4496 (8)	0.1728 (4)	1.1820 (13)	0.114 (5)	
H20A	0.5060	0.1608	1.1910	0.171*	
H20B	0.4269	0.1797	1.2580	0.171*	
H20C	0.4515	0.1999	1.1355	0.171*	
C21	0.3023 (6)	0.2276 (3)	0.9914 (8)	0.053 (2)	
H21A	0.2856	0.2587	1.0019	0.080*	
H21B	0.2778	0.2162	0.9198	0.080*	
H21C	0.3632	0.2258	0.9874	0.080*	
C22	0.2472 (8)	0.2263 (3)	1.1945 (9)	0.089 (4)	
H22A	0.2383	0.2574	1.1737	0.133*	
H22B	0.2912	0.2243	1.2534	0.133*	
H22C	0.1954	0.2139	1.2250	0.133*	
C23	0.1475 (7)	0.1212 (4)	1.1831 (10)	0.090 (4)	
H23A	0.1246	0.1070	1.2518	0.135*	
H23B	0.1331	0.1035	1.1150	0.135*	
H23C	0.1240	0.1511	1.1749	0.135*	
C24	0.2759 (11)	0.1062 (5)	1.3055 (10)	0.131 (6)	
H24A	0.2311	0.0945	1.3534	0.197*	
H24B	0.3047	0.1302	1.3469	0.197*	
H24C	0.3155	0.0825	1.2881	0.197*	
W1	0.717054 (16)	0.272631 (8)	−0.02951 (2)	0.02209 (8)	
Ag1	0.71827 (4)	0.234532 (19)	0.21013 (5)	0.03906 (15)	
S1	0.71534 (12)	0.31578 (6)	0.12878 (15)	0.0323 (4)	
S2	0.71713 (12)	0.19938 (6)	0.01126 (16)	0.0354 (4)	
S3	0.83194 (11)	0.28867 (7)	−0.13097 (17)	0.0370 (4)	
S4	0.60299 (11)	0.28773 (7)	−0.13470 (17)	0.0386 (4)	

### Atomic displacement parameters (Å^2^)

**Table:**

	*U*^11^	*U*^22^	*U*^33^	*U*^12^	*U*^13^	*U*^23^
Lu1	0.02144 (14)	0.01924 (14)	0.03204 (17)	−0.00058 (10)	−0.00007 (12)	−0.00116 (12)
P1	0.0226 (9)	0.0375 (10)	0.0338 (11)	0.0063 (7)	−0.0011 (8)	0.0040 (9)
P2	0.0357 (10)	0.0240 (9)	0.0408 (12)	−0.0047 (7)	0.0058 (9)	−0.0060 (8)
P3	0.0205 (8)	0.0332 (10)	0.0446 (12)	−0.0026 (7)	0.0030 (8)	0.0073 (9)
P4	0.0573 (13)	0.0304 (10)	0.0379 (12)	−0.0103 (9)	−0.0165 (10)	−0.0015 (9)
O1	0.023 (2)	0.033 (3)	0.037 (3)	0.0044 (19)	−0.002 (2)	−0.002 (2)
O2	0.032 (3)	0.023 (2)	0.039 (3)	−0.0048 (19)	−0.001 (2)	−0.004 (2)
O3	0.025 (2)	0.033 (3)	0.046 (3)	−0.0040 (19)	0.007 (2)	0.004 (2)
O4	0.035 (3)	0.026 (2)	0.039 (3)	−0.004 (2)	−0.009 (2)	−0.008 (2)
O5	0.044 (3)	0.027 (3)	0.035 (3)	−0.005 (2)	−0.001 (2)	0.001 (2)
O6	0.043 (3)	0.028 (3)	0.035 (3)	−0.003 (2)	0.003 (2)	−0.001 (2)
O7	0.105 (5)	0.051 (4)	0.039 (4)	−0.015 (3)	−0.015 (3)	0.020 (3)
O8	0.026 (2)	0.032 (3)	0.052 (3)	−0.001 (2)	0.000 (2)	0.007 (2)
O9	0.030 (3)	0.033 (3)	0.045 (3)	−0.003 (2)	0.003 (2)	0.002 (2)
O10	0.058 (4)	0.056 (4)	0.054 (4)	−0.004 (3)	−0.002 (3)	0.029 (3)
N1	0.043 (4)	0.033 (4)	0.044 (4)	−0.001 (3)	−0.002 (3)	0.004 (3)
N2	0.035 (3)	0.026 (3)	0.045 (4)	−0.002 (3)	−0.003 (3)	0.003 (3)
N3	0.027 (3)	0.042 (4)	0.040 (4)	0.001 (3)	0.000 (3)	0.006 (3)
N4	0.036 (4)	0.046 (4)	0.062 (5)	0.006 (3)	0.012 (3)	−0.009 (4)
N5	0.038 (4)	0.066 (5)	0.046 (4)	−0.001 (3)	−0.002 (3)	0.021 (4)
N6	0.043 (4)	0.038 (4)	0.062 (5)	−0.016 (3)	0.015 (3)	−0.020 (3)
N7	0.048 (4)	0.036 (3)	0.038 (4)	−0.007 (3)	0.007 (3)	−0.005 (3)
N8	0.063 (4)	0.030 (3)	0.049 (4)	0.009 (3)	0.012 (4)	0.003 (3)
N9	0.027 (3)	0.060 (4)	0.060 (5)	0.002 (3)	0.004 (3)	0.032 (4)
N10	0.042 (4)	0.067 (5)	0.044 (4)	−0.015 (3)	0.007 (3)	0.007 (4)
N11	0.039 (4)	0.038 (4)	0.085 (6)	−0.007 (3)	0.008 (4)	0.004 (4)
N12	0.079 (6)	0.054 (5)	0.086 (7)	−0.021 (4)	−0.055 (5)	−0.002 (5)
N13	0.096 (6)	0.034 (4)	0.039 (4)	−0.011 (4)	−0.004 (4)	−0.009 (3)
N14	0.104 (7)	0.053 (5)	0.052 (5)	−0.029 (4)	−0.006 (5)	0.006 (4)
C1	0.034 (4)	0.075 (6)	0.052 (6)	0.000 (4)	−0.004 (4)	0.013 (5)
C2	0.036 (4)	0.050 (5)	0.050 (5)	0.001 (3)	0.008 (4)	0.005 (4)
C3	0.064 (6)	0.045 (5)	0.082 (8)	0.020 (4)	0.025 (5)	−0.006 (5)
C4	0.068 (6)	0.046 (5)	0.104 (9)	−0.004 (5)	0.016 (6)	−0.019 (6)
C5	0.064 (7)	0.095 (8)	0.115 (10)	0.012 (6)	−0.007 (6)	0.077 (8)
C6	0.056 (6)	0.171 (12)	0.039 (6)	−0.017 (7)	−0.006 (5)	−0.001 (7)
C7	0.042 (5)	0.057 (6)	0.092 (8)	−0.007 (4)	−0.003 (5)	0.003 (6)
C8	0.093 (8)	0.054 (6)	0.081 (8)	−0.045 (5)	0.030 (6)	−0.023 (6)
C9	0.078 (7)	0.059 (6)	0.042 (5)	−0.006 (5)	0.000 (5)	−0.010 (5)
C10	0.080 (6)	0.045 (5)	0.058 (6)	−0.015 (4)	0.020 (5)	−0.027 (5)
C11	0.105 (9)	0.057 (6)	0.081 (8)	0.028 (6)	0.006 (7)	0.020 (6)
C12	0.065 (7)	0.093 (8)	0.120 (11)	0.024 (6)	0.008 (7)	0.016 (8)
C13	0.034 (5)	0.062 (6)	0.117 (10)	0.007 (4)	0.012 (5)	0.036 (6)
C14	0.026 (4)	0.140 (10)	0.110 (9)	−0.002 (5)	0.006 (5)	0.088 (8)
C15	0.055 (5)	0.068 (6)	0.058 (6)	0.002 (5)	0.003 (5)	−0.005 (5)
C16	0.073 (7)	0.142 (11)	0.060 (7)	−0.042 (7)	0.020 (6)	0.011 (7)
C17	0.116 (10)	0.088 (8)	0.094 (9)	−0.064 (7)	0.046 (8)	−0.049 (7)
C18	0.052 (6)	0.043 (5)	0.158 (12)	0.002 (4)	0.008 (6)	0.049 (7)
C19	0.064 (7)	0.074 (7)	0.119 (11)	0.005 (5)	−0.046 (7)	0.016 (7)
C20	0.123 (11)	0.094 (9)	0.124 (12)	−0.044 (8)	−0.065 (9)	−0.002 (9)
C21	0.080 (6)	0.036 (5)	0.043 (5)	−0.012 (4)	0.007 (5)	0.001 (4)
C22	0.156 (12)	0.055 (6)	0.055 (7)	−0.020 (6)	0.029 (7)	−0.021 (5)
C23	0.110 (9)	0.094 (8)	0.066 (8)	−0.046 (7)	0.038 (7)	−0.011 (6)
C24	0.223 (18)	0.125 (12)	0.045 (7)	0.019 (11)	0.012 (9)	0.041 (8)
W1	0.02831 (14)	0.02062 (14)	0.01730 (14)	0.00242 (10)	−0.00181 (10)	−0.00090 (10)
Ag1	0.0615 (4)	0.0361 (3)	0.0195 (3)	−0.0005 (3)	−0.0008 (3)	0.0021 (2)
S1	0.0505 (11)	0.0232 (9)	0.0231 (9)	−0.0008 (7)	−0.0008 (8)	−0.0063 (7)
S2	0.0579 (12)	0.0204 (8)	0.0280 (10)	0.0045 (8)	−0.0013 (8)	−0.0028 (7)
S3	0.0350 (10)	0.0480 (11)	0.0281 (10)	−0.0060 (8)	0.0023 (8)	−0.0013 (9)
S4	0.0345 (10)	0.0521 (12)	0.0290 (10)	0.0163 (8)	−0.0073 (8)	−0.0039 (9)

### Geometric parameters (Å, º)

**Table:**

Lu1—O4	2.193 (5)	C6—H6A	0.9600
Lu1—O1	2.199 (4)	C6—H6B	0.9600
Lu1—O3	2.210 (4)	C6—H6C	0.9600
Lu1—O2	2.221 (4)	C7—H7A	0.9600
Lu1—O6	2.407 (5)	C7—H7B	0.9600
Lu1—O8	2.429 (5)	C7—H7C	0.9600
Lu1—O9	2.429 (5)	C8—H8A	0.9600
Lu1—O5	2.435 (5)	C8—H8B	0.9600
Lu1—N1	2.838 (7)	C8—H8C	0.9600
Lu1—N2	2.841 (6)	C9—H9A	0.9600
P1—O1	1.497 (4)	C9—H9B	0.9600
P1—N5	1.613 (7)	C9—H9C	0.9600
P1—N4	1.637 (7)	C10—H10A	0.9600
P1—N3	1.640 (6)	C10—H10B	0.9600
P2—O2	1.480 (5)	C10—H10C	0.9600
P2—N8	1.634 (7)	C11—H11A	0.9600
P2—N6	1.635 (6)	C11—H11B	0.9600
P2—N7	1.658 (7)	C11—H11C	0.9600
P3—O3	1.488 (4)	C12—H12A	0.9600
P3—N11	1.615 (7)	C12—H12B	0.9600
P3—N9	1.630 (6)	C12—H12C	0.9600
P3—N10	1.650 (7)	C13—H13A	0.9600
P4—O4	1.482 (5)	C13—H13B	0.9600
P4—N14	1.585 (8)	C13—H13C	0.9600
P4—N13	1.631 (7)	C14—H14A	0.9600
P4—N12	1.635 (8)	C14—H14B	0.9600
O5—N1	1.257 (8)	C14—H14C	0.9600
O6—N1	1.290 (7)	C15—H15A	0.9600
O7—N1	1.219 (8)	C15—H15B	0.9600
O8—N2	1.260 (7)	C15—H15C	0.9600
O9—N2	1.270 (7)	C16—H16A	0.9600
O10—N2	1.225 (8)	C16—H16B	0.9600
N3—C1	1.465 (9)	C16—H16C	0.9600
N3—C2	1.470 (10)	C17—H17A	0.9600
N4—C3	1.452 (10)	C17—H17B	0.9600
N4—C4	1.465 (10)	C17—H17C	0.9600
N5—C6	1.444 (12)	C18—H18A	0.9600
N5—C5	1.477 (11)	C18—H18B	0.9600
N6—C7	1.441 (10)	C18—H18C	0.9600
N6—C8	1.470 (10)	C19—H19A	0.9600
N7—C9	1.442 (11)	C19—H19B	0.9600
N7—C10	1.477 (9)	C19—H19C	0.9600
N8—C11	1.423 (12)	C20—H20A	0.9600
N8—C12	1.455 (11)	C20—H20B	0.9600
N9—C13	1.423 (10)	C20—H20C	0.9600
N9—C14	1.470 (9)	C21—H21A	0.9600
N10—C16	1.452 (10)	C21—H21B	0.9600
N10—C15	1.464 (11)	C21—H21C	0.9600
N11—C18	1.455 (11)	C22—H22A	0.9600
N11—C17	1.468 (13)	C22—H22B	0.9600
N12—C19	1.422 (12)	C22—H22C	0.9600
N12—C20	1.484 (12)	C23—H23A	0.9600
N13—C21	1.459 (10)	C23—H23B	0.9600
N13—C22	1.464 (11)	C23—H23C	0.9600
N14—C23	1.451 (13)	C24—H24A	0.9600
N14—C24	1.479 (14)	C24—H24B	0.9600
C1—H1A	0.9600	C24—H24C	0.9600
C1—H1B	0.9600	W1—S4	2.1917 (19)
C1—H1C	0.9600	W1—S3	2.2034 (18)
C2—H2A	0.9600	W1—S1	2.2059 (17)
C2—H2B	0.9600	W1—S2	2.2105 (17)
C2—H2C	0.9600	W1—Ag1	2.9492 (8)
C3—H3A	0.9600	W1—Ag1^i^	2.9701 (8)
C3—H3B	0.9600	Ag1—S2	2.4890 (19)
C3—H3C	0.9600	Ag1—S1	2.5699 (18)
C4—H4A	0.9600	Ag1—S3^ii^	2.621 (2)
C4—H4B	0.9600	Ag1—S4^ii^	2.625 (2)
C4—H4C	0.9600	Ag1—W1^ii^	2.9701 (8)
C5—H5A	0.9600	S3—Ag1^i^	2.621 (2)
C5—H5B	0.9600	S4—Ag1^i^	2.625 (2)
C5—H5C	0.9600		
			
O4—Lu1—O1	93.25 (17)	N5—C5—H5C	109.5
O4—Lu1—O3	89.36 (18)	H5A—C5—H5C	109.5
O1—Lu1—O3	156.72 (17)	H5B—C5—H5C	109.5
O4—Lu1—O2	157.54 (18)	N5—C6—H6A	109.5
O1—Lu1—O2	92.89 (16)	N5—C6—H6B	109.5
O3—Lu1—O2	93.46 (17)	H6A—C6—H6B	109.5
O4—Lu1—O6	129.22 (17)	N5—C6—H6C	109.5
O1—Lu1—O6	79.56 (17)	H6A—C6—H6C	109.5
O3—Lu1—O6	80.90 (18)	H6B—C6—H6C	109.5
O2—Lu1—O6	73.18 (16)	N6—C7—H7A	109.5
O4—Lu1—O8	78.87 (17)	N6—C7—H7B	109.5
O1—Lu1—O8	127.65 (16)	H7A—C7—H7B	109.5
O3—Lu1—O8	75.54 (16)	N6—C7—H7C	109.5
O2—Lu1—O8	80.28 (17)	H7A—C7—H7C	109.5
O6—Lu1—O8	143.11 (16)	H7B—C7—H7C	109.5
O4—Lu1—O9	79.69 (17)	N6—C8—H8A	109.5
O1—Lu1—O9	75.17 (17)	N6—C8—H8B	109.5
O3—Lu1—O9	127.97 (17)	H8A—C8—H8B	109.5
O2—Lu1—O9	81.05 (17)	N6—C8—H8C	109.5
O6—Lu1—O9	142.66 (16)	H8A—C8—H8C	109.5
O8—Lu1—O9	52.48 (15)	H8B—C8—H8C	109.5
O4—Lu1—O5	76.22 (18)	N7—C9—H9A	109.5
O1—Lu1—O5	78.13 (16)	N7—C9—H9B	109.5
O3—Lu1—O5	80.06 (16)	H9A—C9—H9B	109.5
O2—Lu1—O5	126.22 (17)	N7—C9—H9C	109.5
O6—Lu1—O5	53.04 (16)	H9A—C9—H9C	109.5
O8—Lu1—O5	145.06 (16)	H9B—C9—H9C	109.5
O9—Lu1—O5	142.63 (16)	N7—C10—H10A	109.5
O4—Lu1—N1	102.39 (19)	N7—C10—H10B	109.5
O1—Lu1—N1	76.47 (17)	H10A—C10—H10B	109.5
O3—Lu1—N1	80.37 (18)	N7—C10—H10C	109.5
O2—Lu1—N1	100.05 (18)	H10A—C10—H10C	109.5
O6—Lu1—N1	26.90 (17)	H10B—C10—H10C	109.5
O8—Lu1—N1	155.87 (16)	N8—C11—H11A	109.5
O9—Lu1—N1	151.64 (17)	N8—C11—H11B	109.5
O5—Lu1—N1	26.18 (16)	H11A—C11—H11B	109.5
O4—Lu1—N2	75.86 (18)	N8—C11—H11C	109.5
O1—Lu1—N2	101.52 (17)	H11A—C11—H11C	109.5
O3—Lu1—N2	101.54 (17)	H11B—C11—H11C	109.5
O2—Lu1—N2	81.75 (17)	N8—C12—H12A	109.5
O6—Lu1—N2	154.92 (16)	N8—C12—H12B	109.5
O8—Lu1—N2	26.21 (15)	H12A—C12—H12B	109.5
O9—Lu1—N2	26.44 (15)	N8—C12—H12C	109.5
O5—Lu1—N2	152.00 (17)	H12A—C12—H12C	109.5
N1—Lu1—N2	177.33 (18)	H12B—C12—H12C	109.5
O1—P1—N5	110.6 (3)	N9—C13—H13A	109.5
O1—P1—N4	111.8 (3)	N9—C13—H13B	109.5
N5—P1—N4	106.8 (4)	H13A—C13—H13B	109.5
O1—P1—N3	108.6 (3)	N9—C13—H13C	109.5
N5—P1—N3	109.6 (3)	H13A—C13—H13C	109.5
N4—P1—N3	109.4 (3)	H13B—C13—H13C	109.5
O2—P2—N8	117.9 (3)	N9—C14—H14A	109.5
O2—P2—N6	108.9 (3)	N9—C14—H14B	109.5
N8—P2—N6	104.0 (4)	H14A—C14—H14B	109.5
O2—P2—N7	107.7 (3)	N9—C14—H14C	109.5
N8—P2—N7	103.7 (3)	H14A—C14—H14C	109.5
N6—P2—N7	114.8 (3)	H14B—C14—H14C	109.5
O3—P3—N11	119.9 (3)	N10—C15—H15A	109.5
O3—P3—N9	107.8 (3)	N10—C15—H15B	109.5
N11—P3—N9	104.5 (4)	H15A—C15—H15B	109.5
O3—P3—N10	107.5 (3)	N10—C15—H15C	109.5
N11—P3—N10	102.7 (4)	H15A—C15—H15C	109.5
N9—P3—N10	114.7 (4)	H15B—C15—H15C	109.5
O4—P4—N14	111.0 (4)	N10—C16—H16A	109.5
O4—P4—N13	109.9 (3)	N10—C16—H16B	109.5
N14—P4—N13	108.2 (4)	H16A—C16—H16B	109.5
O4—P4—N12	109.6 (4)	N10—C16—H16C	109.5
N14—P4—N12	108.5 (5)	H16A—C16—H16C	109.5
N13—P4—N12	109.6 (4)	H16B—C16—H16C	109.5
P1—O1—Lu1	168.0 (3)	N11—C17—H17A	109.5
P2—O2—Lu1	161.0 (3)	N11—C17—H17B	109.5
P3—O3—Lu1	158.9 (3)	H17A—C17—H17B	109.5
P4—O4—Lu1	168.3 (3)	N11—C17—H17C	109.5
N1—O5—Lu1	95.1 (4)	H17A—C17—H17C	109.5
N1—O6—Lu1	95.5 (4)	H17B—C17—H17C	109.5
N2—O8—Lu1	95.5 (4)	N11—C18—H18A	109.5
N2—O9—Lu1	95.2 (4)	N11—C18—H18B	109.5
O7—N1—O5	122.9 (6)	H18A—C18—H18B	109.5
O7—N1—O6	120.9 (7)	N11—C18—H18C	109.5
O5—N1—O6	116.1 (6)	H18A—C18—H18C	109.5
O7—N1—Lu1	175.0 (5)	H18B—C18—H18C	109.5
O5—N1—Lu1	58.7 (3)	N12—C19—H19A	109.5
O6—N1—Lu1	57.6 (3)	N12—C19—H19B	109.5
O10—N2—O8	122.3 (6)	H19A—C19—H19B	109.5
O10—N2—O9	121.5 (6)	N12—C19—H19C	109.5
O8—N2—O9	116.2 (6)	H19A—C19—H19C	109.5
O10—N2—Lu1	172.6 (5)	H19B—C19—H19C	109.5
O8—N2—Lu1	58.3 (3)	N12—C20—H20A	109.5
O9—N2—Lu1	58.4 (3)	N12—C20—H20B	109.5
C1—N3—C2	115.0 (6)	H20A—C20—H20B	109.5
C1—N3—P1	122.0 (5)	N12—C20—H20C	109.5
C2—N3—P1	119.2 (5)	H20A—C20—H20C	109.5
C3—N4—C4	113.9 (7)	H20B—C20—H20C	109.5
C3—N4—P1	125.4 (6)	N13—C21—H21A	109.5
C4—N4—P1	119.9 (6)	N13—C21—H21B	109.5
C6—N5—C5	115.4 (9)	H21A—C21—H21B	109.5
C6—N5—P1	119.5 (7)	N13—C21—H21C	109.5
C5—N5—P1	123.6 (7)	H21A—C21—H21C	109.5
C7—N6—C8	113.2 (7)	H21B—C21—H21C	109.5
C7—N6—P2	121.3 (6)	N13—C22—H22A	109.5
C8—N6—P2	120.4 (6)	N13—C22—H22B	109.5
C9—N7—C10	114.0 (7)	H22A—C22—H22B	109.5
C9—N7—P2	120.2 (5)	N13—C22—H22C	109.5
C10—N7—P2	119.5 (5)	H22A—C22—H22C	109.5
C11—N8—C12	109.9 (8)	H22B—C22—H22C	109.5
C11—N8—P2	124.3 (6)	N14—C23—H23A	109.5
C12—N8—P2	121.3 (6)	N14—C23—H23B	109.5
C13—N9—C14	113.3 (6)	H23A—C23—H23B	109.5
C13—N9—P3	122.7 (5)	N14—C23—H23C	109.5
C14—N9—P3	121.2 (5)	H23A—C23—H23C	109.5
C16—N10—C15	113.0 (8)	H23B—C23—H23C	109.5
C16—N10—P3	120.8 (6)	N14—C24—H24A	109.5
C15—N10—P3	119.0 (6)	N14—C24—H24B	109.5
C18—N11—C17	112.7 (8)	H24A—C24—H24B	109.5
C18—N11—P3	123.8 (7)	N14—C24—H24C	109.5
C17—N11—P3	120.4 (7)	H24A—C24—H24C	109.5
C19—N12—C20	112.6 (9)	H24B—C24—H24C	109.5
C19—N12—P4	122.6 (6)	S4—W1—S3	109.89 (7)
C20—N12—P4	124.2 (8)	S4—W1—S1	108.20 (7)
C21—N13—C22	115.8 (7)	S3—W1—S1	108.67 (7)
C21—N13—P4	118.8 (6)	S4—W1—S2	108.19 (8)
C22—N13—P4	123.0 (7)	S3—W1—S2	108.73 (7)
C23—N14—C24	115.6 (9)	S1—W1—S2	113.13 (7)
C23—N14—P4	120.0 (7)	S4—W1—Ag1	125.49 (6)
C24—N14—P4	124.4 (9)	S3—W1—Ag1	124.61 (6)
N3—C1—H1A	109.5	S1—W1—Ag1	57.67 (5)
N3—C1—H1B	109.5	S2—W1—Ag1	55.48 (5)
H1A—C1—H1B	109.5	S4—W1—Ag1^i^	58.85 (5)
N3—C1—H1C	109.5	S3—W1—Ag1^i^	58.65 (6)
H1A—C1—H1C	109.5	S1—W1—Ag1^i^	148.83 (5)
H1B—C1—H1C	109.5	S2—W1—Ag1^i^	98.03 (5)
N3—C2—H2A	109.5	Ag1—W1—Ag1^i^	153.503 (12)
N3—C2—H2B	109.5	S2—Ag1—S1	93.51 (6)
H2A—C2—H2B	109.5	S2—Ag1—S3^ii^	121.16 (6)
N3—C2—H2C	109.5	S1—Ag1—S3^ii^	120.13 (6)
H2A—C2—H2C	109.5	S2—Ag1—S4^ii^	120.53 (7)
H2B—C2—H2C	109.5	S1—Ag1—S4^ii^	117.74 (6)
N4—C3—H3A	109.5	S3^ii^—Ag1—S4^ii^	86.61 (6)
N4—C3—H3B	109.5	S2—Ag1—W1	47.03 (4)
H3A—C3—H3B	109.5	S1—Ag1—W1	46.49 (4)
N4—C3—H3C	109.5	S3^ii^—Ag1—W1	137.38 (5)
H3A—C3—H3C	109.5	S4^ii^—Ag1—W1	135.95 (5)
H3B—C3—H3C	109.5	S2—Ag1—W1^ii^	151.28 (5)
N4—C4—H4A	109.5	S1—Ag1—W1^ii^	115.17 (4)
N4—C4—H4B	109.5	S3^ii^—Ag1—W1^ii^	45.90 (4)
H4A—C4—H4B	109.5	S4^ii^—Ag1—W1^ii^	45.61 (4)
N4—C4—H4C	109.5	W1—Ag1—W1^ii^	161.66 (2)
H4A—C4—H4C	109.5	W1—S1—Ag1	75.84 (5)
H4B—C4—H4C	109.5	W1—S2—Ag1	77.49 (5)
N5—C5—H5A	109.5	W1—S3—Ag1^i^	75.45 (6)
N5—C5—H5B	109.5	W1—S4—Ag1^i^	75.54 (6)
H5A—C5—H5B	109.5		

Symmetry codes: (i) *x*, −*y*+1/2, *z*−1/2; (ii) *x*, −*y*+1/2, *z*+1/2.
